# Differential functional roles of fibroblasts and pericytes in the formation of tissue-engineered microvascular networks in vitro

**DOI:** 10.1038/s41536-019-0086-3

**Published:** 2020-01-06

**Authors:** Natalia Kosyakova, Derek D. Kao, Maria Figetakis, Francesc López-Giráldez, Susann Spindler, Morven Graham, Kevin J. James, Jee Won Shin, Xinran Liu, Gregory T. Tietjen, Jordan S. Pober, William G. Chang

**Affiliations:** 1 Department of Medicine, Section of Nephrology, Yale University School of Medicine, New Haven, CT 06520 USA; 2 Yale College of Undergraduate Studies, Yale University, New Haven, CT 06520 USA; 3 Yale Center for Genome Analysis, Yale University School of Medicine, Orange, CT 06477 USA; 4 Department of Biomedical Engineering, Yale University School of Medicine, New Haven, CT 06520 USA; 5 Yale Center for Cellular and Molecular Imaging, Yale University School of Medicine, New Haven, CT 06510 USA; 6 Department of Immunobiology, Yale University School of Medicine, New Haven, CT 06519 USA

**Keywords:** Tissue engineering, Cell adhesion, Gene expression, Regenerative medicine, Cell biology

## Abstract

Formation of a perfusable microvascular network (μVN) is critical for tissue engineering of solid organs. Stromal cells can support endothelial cell (EC) self-assembly into a μVN, but distinct stromal cell populations may play different roles in this process. Here we describe the differential effects that two widely used stromal cell populations, fibroblasts (FBs) and pericytes (PCs), have on μVN formation. We examined the effects of adding defined stromal cell populations on the self-assembly of ECs derived from human endothelial colony forming cells (ECFCs) into perfusable μVNs in fibrin gels cast within a microfluidic chamber. ECs alone failed to fully assemble a perfusable μVN. Human lung FBs stimulated the formation of EC-lined μVNs within microfluidic devices. RNA-seq analysis suggested that FBs produce high levels of hepatocyte growth factor (HGF). Addition of recombinant HGF improved while the c-MET inhibitor, Capmatinib (INCB28060), reduced μVN formation within devices. Human placental PCs could not substitute for FBs, but in the presence of FBs, PCs closely associated with ECs, formed a common basement membrane, extended microfilaments intercellularly, and reduced microvessel diameters. Different stromal cell types provide different functions in microvessel assembly by ECs. FBs support μVN formation by providing paracrine growth factors whereas PCs directly interact with ECs to modify microvascular morphology.

## Introduction

Microvascular network (μVN) formation is critically important for tissue engineering of organs too thick to be maintained by diffusive nutrient transport alone. We and others have generated human EC-derived microvascular networks (μVNs) in vivo within gels implanted into immunodeficient mice.^1^ However, human ECs suspended in the same gels in vitro initially assemble into cords but fail to fully form a μVN as the cells typically die between 24 and 36 h. To improve vascularization, in previous experiments, we have overexpressed Bcl-2 to reduce the apoptotic response of human umbilical vein endothelial cells (HUVEC) in collagen/fibronectin matrices.^2,3^ More recently, we have utilized human ECs differentiated from human endothelial colony forming cell (ECFCs) that also form vessels in vivo and have much greater replicative life spans than HUVECs, an important advantage for tissue engineering.^4^ However, like HUVECs, untransduced ECFC failed to form stable μVNs in vitro. Although Bcl-2 overexpression does not seem to cause transformation or give rise to tumors in vivo, there is still concern about this approach in clinically implanted tissues.

Microvessels are normally surrounded by extracellular matrix, stromal FBs, and supporting PCs that are intimately associated with the endothelium and share a common basement membrane. FBs are believed to be the principal cells of stromal tissue with critical roles in synthesis of extracellular matrix. FBs have key roles in the development and morphogenesis of tissues and organs.^5^ In contrast, PCs are critical for vascular development and for stabilization of the microcirculation. They are thought to regulate vascular tone, permeability, and have immunological functions.^6^ Genetic or acquired deficiencies in PC coverage of endothelial-lined capillaries result in abnormal microvasculature characterized by increased microvessel diameter and increased permeability.^7–9^

Thus, important biological questions arise about the roles of stromal cell types such as FBs and PCs in successful microvascular tissue engineering. In previous studies, FBs have been shown to support EC sprouting and lumen formation after being seeded onto collagen coated dextran beads within 3D fibrin gels. Secretion of FB factors is thought to be important in this angiogenic response.^10,11^ We have been using human PCs derived from post-partum placental tissue. We believe that these readily attainable cells are very useful for microvascular engineering applications. We have observed that cultured human placental PCs invest tissue-engineered human microvasculature when implanted in vivo,^12,13^ and that the presence of human PCs led to mural coverage, decreased vessel size, and permeability in tissue-engineered microvessels.^12^

Therefore, we believe that host stromal and EC interactions are critically important for the formation of μVNs. This is supported by observations that non-transduced human ECs formed robust μVNs when co-implanted with human mesenchymal stem cells,^14^ human lung FBs,^15,16^ or mouse 10T1/2 cells.^17^ Others have described that human lung FBs can support EC survival and μVN formation in fibrin gels within a microfluidic device.^8,9^ These concepts and previous experimental investigations prompted us to compare the differential functions of FBs and PCs on ECFC-derived microvascular networks in an in vitro microfluidic chamber containing cells suspended in fibrin hydrogels. Here we report that these two stromal cell types are indeed distinct and play very different roles in μVN formation.

## Results

### Co-culture of ECs and FBs within microfluidic devices

**Fig. 1:**
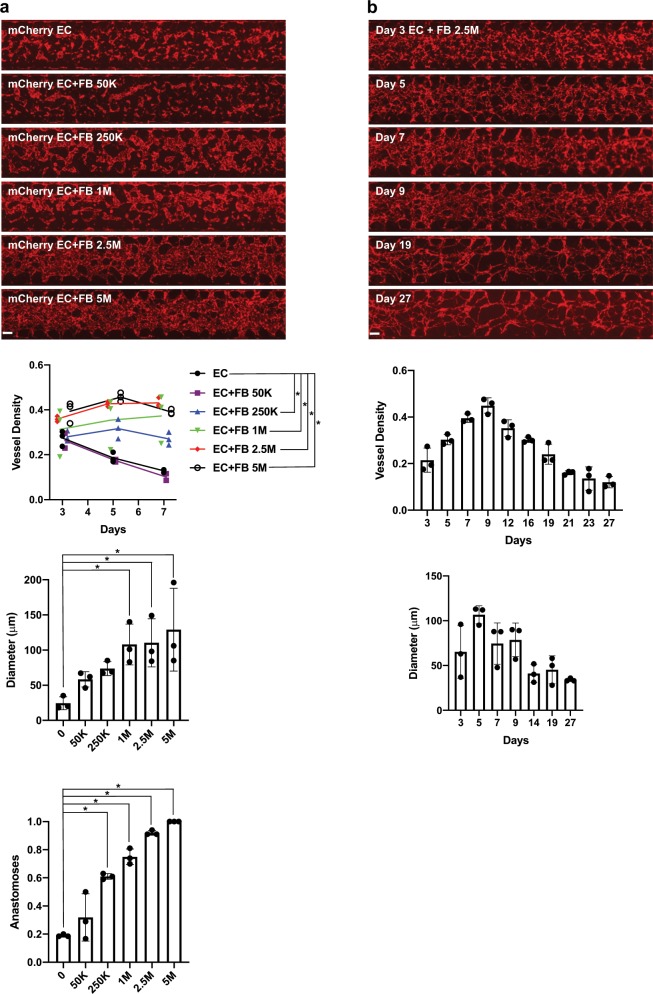
FBs stabilize μVNs. **a** Microfluidic devices with FB titration cultured for 7 days. EC concentrations were 1.0 × 10^7^ cells per ml and FBs ranged from 5.0 × 10^5^ to 5.0 × 10^6^. Graphs quantify vessel density, diameter, and anastomoses within microfluidic devices. Statistically significant differences between groups are indicated by asterisks (*). **b** Long-term culture of microfluidic devices with 1.0 × 10^7^ ECFCs per ml and 2.5 × 10^6^ per ml FBs. Graphs quantify vessel density and diameters over the long time-course. Scale bars of tile images are 250 µm.

In previous studies, we have observed that implantation of ECFCs alone in vivo leads to formation of robust μVNs.^4,18^ However, within microfluidic devices, ECFCs alone are not sufficient to form μVNs (Fig. 1a). Indeed, ECFC cords started to emerge by day 3, but began to deteriorate by day 5 and never achieved perfusable networks. Next, we investigated whether addition of a commonly utilized, commercially available normal human lung FBs could improve μVN formation. We found that a minimum of 2.5 × 10^5^ FBs per ml were needed to improve μVN density. Furthermore, microvessel diameter and anastomoses with the inlet and outlet pores also increased with addition of FBs (Fig. 1a). In subsequent experiments, we used 2.5 × 10^6^ FBs per ml of matrix when co-cultured with ECFCs. We confirmed that μVNs were perfusable in the ECFC and FB co-cultured devices by flowing fluorescently labeled beads through the channels (Supplementary Movie 1). Although most experiments we performed were in cultured devices for 7 days, we wanted to see how μVNs behaved in long-term culture. We thus observed the morphology of μVNs derived from co-cultured ECFCs with FBs for 27 days in the microfluidic devices. Beyond 9 days, microvessel density and diameters declined (Fig. 1b).

### Co-culture of PC modified microvessel diameters

**Fig. 2:**
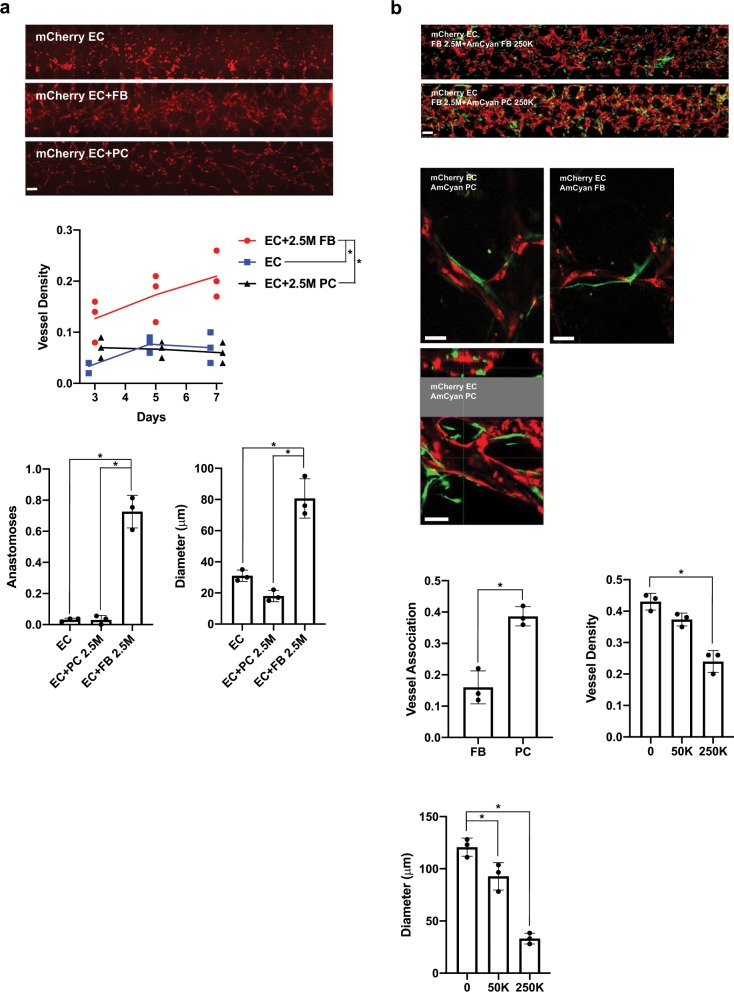
PCs are closely associated with and reduce the lumen diameters of μVNs. **a** Microfluidic devices cultured for 7 days with 1.0 × 10^7^ ECFCs per ml or ECFCs with 2.5 × 10^6^ per ml PCs or FBs. Graphs quantify vessel density, anastomoses, and vessel diameters. **b** (Top) Tiled epifluorescence images of microfluidic devices cultured for 7 days with 1.0 × 10^7^ ECFCs, 2.5 × 10^6^ FBs, and 2.5 × 10^5^ AmCyan FBs or 2.5 × 10^5^ per ml of AmCyan PCs. (Middle) Confocal microscopy of EC-PC and EC-FB interactions. Second EC-PC image contains z-stack through the microvessel showing interaction of PCs with ECs through thickness of a single microvessel. (Bottom) Graphs quantify stromal-EC association (after analysis by confocal microscopy and z-stacks), vessel density, and diameters with PC titration. In all microscopy, EC express mCherry (red) and PCs or FBs express AmCyan (green) as indicated. Tiled image scale bars are 250 µm and confocal image scale bars are 75 µm. Statistically significant differences between groups are indicated by asterisks (*).

Given that PCs are known to stabilize microvessels in vivo, we investigated whether PCs co-cultured with ECFCs could also support the formation and maintenance of microvessels. Surprisingly, unlike FBs, PCs co-cultured with ECFCs did not yield stable μVNs. Vessel density, anastomoses, and diameter were significantly reduced when FBs were not included, even when PCs were added at the same concentration (Fig. 2a).

Having observed that ECFCs and PCs were not sufficient to form stable μVNs, we co-cultured PCs with ECFCs and FBs to examine the effects of PCs on the μVNs (Fig. 2b). We observed that the addition of PCs reduced the diameters of the microvessels formed. The mean diameter of the microvessels with no PC incorporation was 120 μm, however, the mean diameter of μVNs with 2.5 × 10^5^ PCs per ml were 33 μm. In addition, there was an observable dose effect as diameters decreased with increasing PC number. We also observed that PCs were more closely associated with the microvessels formed than FBs when we examined μVNs by confocal microscopy. Fifty AmCyan fluorescently labeled FBs or PCs were individually examined by confocal microscopy and were identified as either associated with mCherry labeled microvessel or not associated after examination of serial sections of a z-stack. Of the counted cells, 16% of FBs were associated with microvessels, while 39% of PCs were associated with microvessels. PCs were significantly more likely to be associated with microvessels.

### RNA-seq comparison of FBs and PCs

**Fig. 3:**
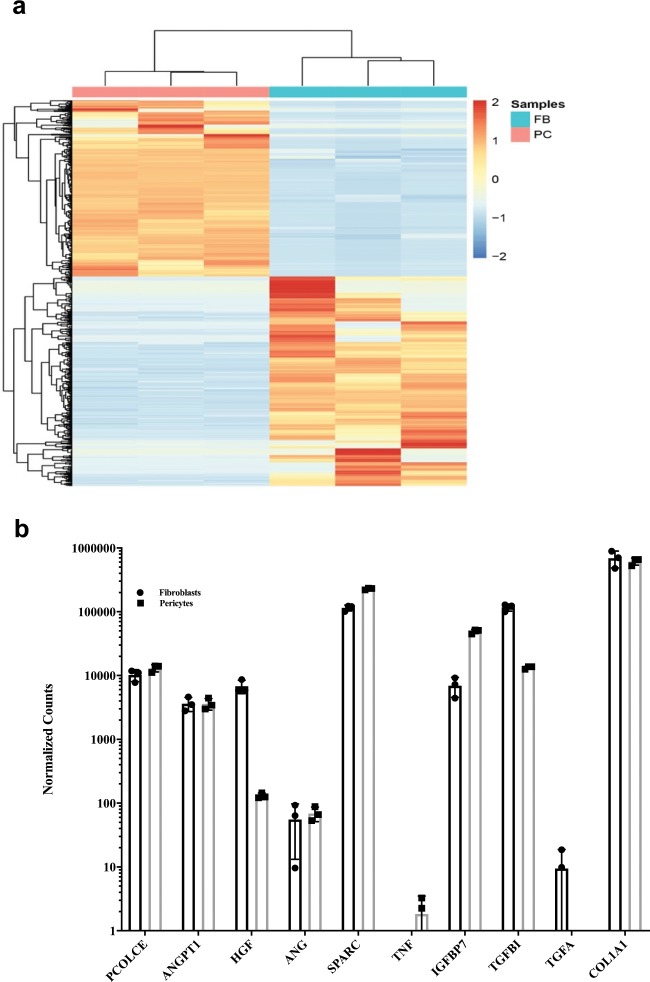
RNA-seq analysis of FBs versus PCs. **a** Heatmap of the gene expression profiles of FBs and PCs. Genes significantly differentially expressed (*q*-value < 0.05) with a log_2_[fold change]≥5 between FBs and PCs are shown (*n* = 1056). Cells from individual donors are represented in each column. Each row represents gene expressions across both cell types. Rows and columns are hierarchically clustered. Expression values are scaled by row; red color indicates higher gene expression and blue indicates lower gene expression. **b** Regularized log counts with standard deviations of FB factors previously shown to have positive effects on microvessel formation.

Given the differences that we observed within the microfluidic devices when ECFCs were co-cultured with FBs or PCs, we used RNA-seq to compare bulk gene expression profiles of the FBs and PCs. We prepared and analyzed FB gene expression profiles with RNA-seq using three different donors in the same manner as previously published for PCs^19^ (Supplementary Data 1). We identified 1056 genes that were differentially expressed (*q*-value < 0.05 and log_2_[fold change]≥5; Fig. 3a). Of the differentially expressed genes, we searched for proteins that may contribute to the phenotypic differences that we observed between FBs and PCs. In static endothelial sprouting models, others have identified factors that contribute both to vessel sprouting (angiopoietin-1, angiogenin, HGF, transforming growth factor-α, and tumor necrosis factor) and lumen formation (collagen I, procollagen C endopeptidase enhancer 1, secreted protein acidic and rich in cysteine [SPARC], transforming growth factor-β induced protein ig-h3 [βig-h3], and insulin growth-binding protein 7).^10^ When we specifically profiled the FB factors previously reported to enhance microvessel formation,^10^ we observed that HGF and βig-h3 or transforming growth factor β induced (TGFBI) as it is now named were significantly upregulated in FBs (Fig. 3b). Collagen I expression was high in both FBs and PCs cultured as monolayers. Given the close association of PCs to ECFCs within the μVNs, we looked at subsets of genes involved in cell–cell, cell–matrix adhesion, and extracellular matrix proteins using gene ontology (GO0031012, GO0007160, and GO0098609). We observed that several integrin subunits, matrix adhesion genes, and basement membrane proteins were upregulated in PCs (Supplementary Table and Supplementary Figures 3–5). Of extracellular matrix genes (GO0031012), PCs upregulated *COL4A1*, *COL4A2*, *COL4A5*, *SPARC*, and *LAMA5*. Of cell–matrix adhesion genes (GO0007160), PCs upregulated *ITGA1*, *ITGA3*, *ITGA4*, *ITGA6*, *ITGA8*, *ITGA11*, *ITGAV*, *ITGB5*, and *L1CAM*. Of cell–cell adhesion genes (GO0098609), PCs upregulated *DSP*, *ESAM*, and *ICAM1*. Collectively, these data suggest that PCs differ from FBs in their expression of extracellular matrix, cell–matrix adhesion, and cell–cell adhesion genes*.* These transcriptional differences likely contribute to the phenotypic differences that we observed between these cells within the microfluidic devices.

### HGF stimulates μVNs in microfluidic devices

**Fig. 4:**
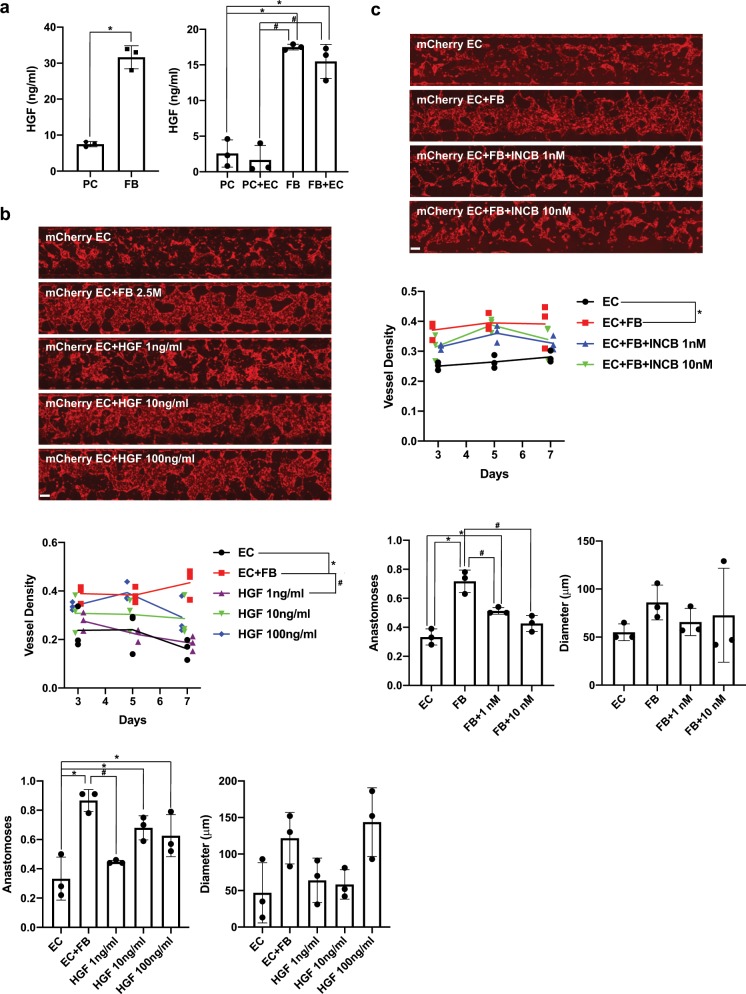
HGF stimulates μVNs. **a** FBs secrete more HGF than PCs as measured by ELISA quantification. Co-culture of ECs in vitro does not affect HGF secretion by PCs or FBs. **b** Tiled images of microfluidic devices containing ECs without and with FBs or rHGF added to the media (at indicated concentrations). Graphs indicate vessel density, anastomoses, and mean diameters. **c** Tiled images of microfluidic devices containing ECs without and with FBs or c-Met inhibitor INCB28060 at the indicated concentrations. Graphs quantify vessel density, anastomoses, and mean diameters. Scale bars for tiled images are 250 µm. Asterisks (*) and hashes (#) indicate statistical differences between indicated groups.

While a complete analysis of genes differentially expressed by FBs and PCs is beyond the scope of this current series of experiments, we did seek to establish whether the microfluidic devices could be used as a tool for dissecting how FBs contribute to stabilization of μVNs. Of FB factors reported to stimulate microvessel formation in vitro, we observed that HGF was significantly higher in FBs than in PCs. HGF has been demonstrated to stimulate blood vessel formation^20,21^ and has important roles in development, cell survival, and tissue regeneration via binding and activation of its receptor, c-MET.^22^ *HGF* had an ~50-fold higher gene expression in FBs than PCs. We observed by ELISA that ~5–7-fold higher levels of HGF protein was secreted by FBs than PCs (Fig. 4a). However, we did not see that co-culture of ECFCs with FB or PC in vitro improved HGF secretion. When rHGF was added to the culture media of the microfluidic devices, we saw a trend toward improved density and diameter, but these differences were not statistically significant. We did observe a statistically significant improvement in the anastomoses with the inlet and outlet pores with rHGF addition (Fig. 4b). We also observed that 1 and 10 nM of the c-MET kinase inhibitor, INCB28060 (Capmatinib), only partially reduced μVN density (Fig. 4c), yet reduced anastomoses. Overall, these data suggest that HGF has a stimulatory effect, but FBs likely contribute other factors important to μVN formation and stability, including the sprouting and lumen formation factors cited above.^10^

### Ultrastructural and basement membrane analyses of μVNs

**Fig. 5:**
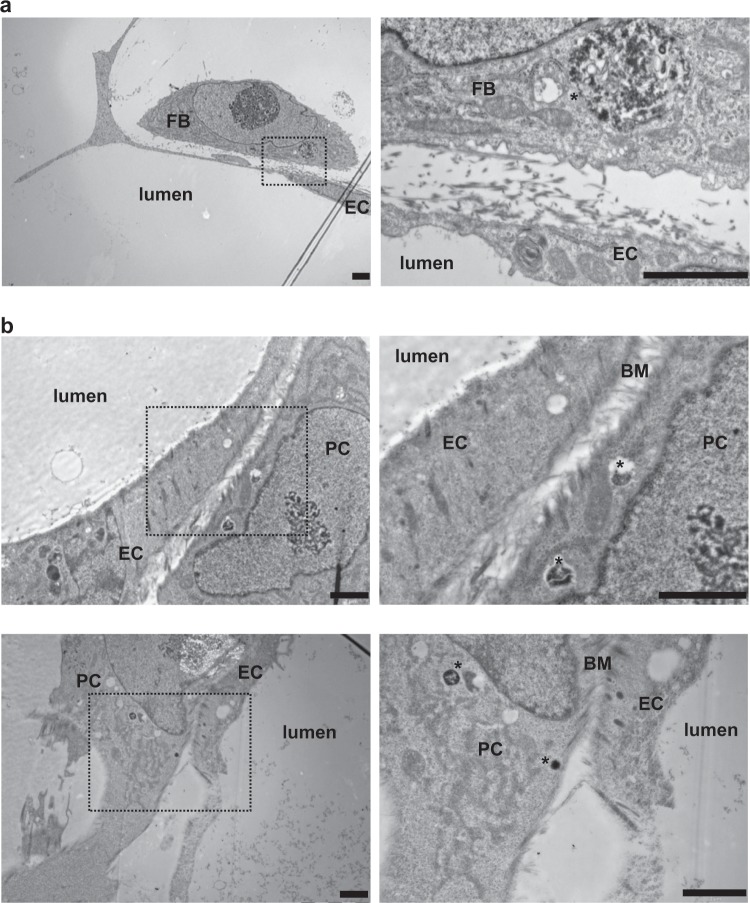
Ultrastructural analysis of cell–cell interactions within μVNs. PCs and FBs were pre-labeled with Molday ION particles prior to formation of μVNs containing EC, FBs, and iron-labeled FBs (**a**) or iron-labeled PCs (**b**). Devices were analyzed by TEM after 7 days. **a** Demonstrates loose association between EC and FB. Inset shows iron-labeled endosome indicated by asterisk (*) within FB and collagen fibers between EC and FB. **b** Demonstrates close interaction between EC and iron-labeled PC. Insets show dynamic interaction observed between EC and PC and highlight iron-labeled endosomes (indicated by *) and common basement between two interacting cells. Scale bars are 2 µm.

**Fig. 6:**
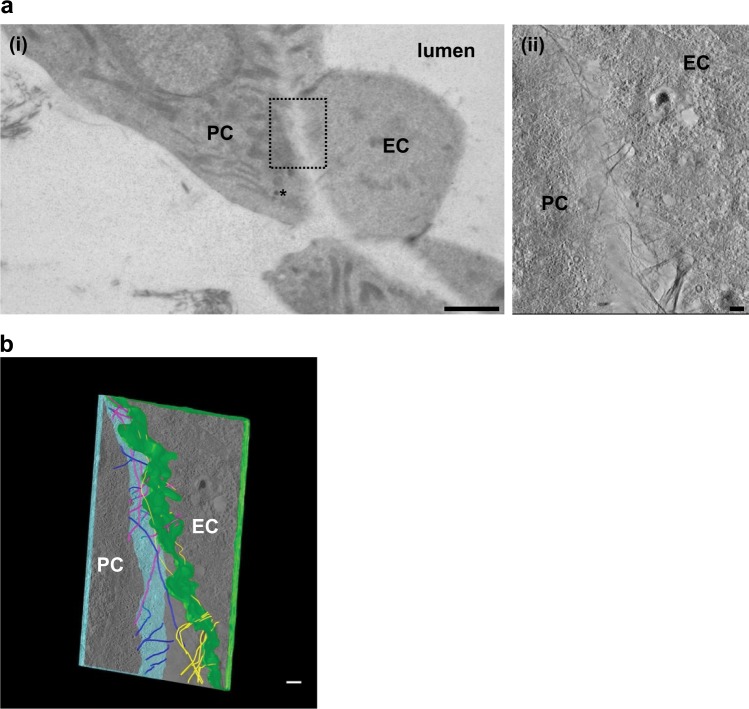
Electron tomography of EC and PC interaction. **a** Visualization of the field used to create tomography slices. Higher magnification area indicates the area which tomographic slices were created from and modeled using iMOD software (inset). Inset shows iron-labeled endosome indicated by asterisks (*). **b** 3D model reconstruction of tomography slices tracking filaments through the z-stack to demonstrate how filaments were oriented between the two cells. EC border was outlined in green and PC in blue. Microfilaments are outlined in three colors: dark blue, magenta, and yellow to distinguish the location filaments originate from. Dark blue filaments originate in PC and yellow filaments originate in ECFC and do not cross over into neighboring cell. Magenta filaments connect both cell types. Scale bars are 2 µm (**a**(i)) and 200 nm (**a**(ii), **b**).

To further investigate the differences between PC and FB interactions within microvascular networks, we performed transmission electron microscopy (TEM) on the μVNs within the microfluidic devices at the 7-day time point. To distinguish the different cell types, we pre-labeled FBs and PCs with Molday ION (iron oxide particles). These particles accumulate in endosomes and are readily detected in TEM as electron dense particles (Supplementary Figure 6) within endosomes.^13^ With TEM, we rarely observed occurrences where the ECs and FBs were closely associated (Fig. 5a). When we did see associations, the cells remained distinct with the presence of collagen fibers between them. However, there was a much more dynamic interaction between ECs and PCs with formation of microfilaments between the two. In some cases, both cell types appeared to protrude actin filaments towards each other. In addition, we observed instances where a more established common basement membrane had formed (Fig. 5b). To better analyze the interaction between ECs and PCs, we used electron tomography to obtain a 3D delineation of the boundary between these cells (Fig. 6 and Supplementary Movie 2). We observed that both cell types appeared to extend microfilaments towards each other, some were shared by the two cells and in specific places, cells were able to contact each other.

Our RNA-seq data and previously reported descriptions of PC effects^23^ on microvascular networks suggested that PCs may be contributing collagen IV to the basement membrane of the μVNs. However, when we looked by IF and confocal microscopy at collagen IV deposition in the μVNs within the microfluidic devices, we saw that ECs themselves deposited collagen IV that was not enhanced by the co-culture of PCs (Supplementary Figure 7) at 1 week of culture. Interestingly, at 2 weeks of culture, we observed wide staining of collagen IV around the remaining vessels, with PCs remaining adjacent to the ECs, but within the collagen IV area. This is consistent with prior observations that PCs reside within the basement membrane shared with ECs in vivo. It is possible that immunofluorescence and confocal microscopy lacks the resolution necessary to see the additive effect of collagen IV deposited by PCs.

## Discussion

A better understanding of how μVNs form is critical to advancing tissue engineering and future regenerative medicine approaches. However, the assembly of μVNs is a complex process with multiple cell–cell, cell–matrix, and signaling pathways necessary for success. Here we have examined how two different stromal cell types, FBs and PCs, affect formation of μVNs. We postulate that FBs sustain and stimulate μVN formation through paracrine release of survival factors, while PCs directly contact ECFCs to modify microvessel morphology.

A key issue raised in this study is the type of cells to use for tissue engineering research of μVNs. The same types of cells from different tissue sources may significantly vary in their behaviors. We chose human ECs derived from cord blood ECFCs, human lung FBs, and human placental PCs. We selected this EC population because it displays extended replicative lifespan and robust vasculogenic potential, is readily obtained, and can be genetically modified and clonally selected after CRISPR/Cas9 implementation.^4^ We selected human lung FBs because of their wide-spread use in experimental settings of tissue engineering. However, an interesting extension of this work would be to examine dermal FBs, which might have similar μVN stabilizing properties, and from a clinical perspective be more readily obtained. Indeed, in a previous study, dermal FBs were found to stimulate EC sprouting in a microfluidic model.^24^ In that study, dermal FBs facilitated EC-PC associations, while lung FBs were not conducive to EC-PC associations. This differs from our findings that EC-PC associate in the presence of lung FBs co-culture. In that investigation, FBs were separated from the ECs and PCs to promote sprouting into a hydrogel. This may indicate that spatial gradients of factors secreted by FBs might also influence EC-PC interactions. Finally, we have chosen placental PCs because they are also readily obtained from discarded post-partum tissue and have been characterized both behaviorally and by transcriptomics.^19^ We analyzed the interactions of these cells within gels cast in microfluidic chambers because this in vitro setting gives rise to perfusable μVNs, better recapitulating the in vivo setting.

The RNA-seq analyses demonstrate that FBs and PCs possess distinct gene expression patterns that likely contribute to the differences in their effects on μVNs within these devices. Looking at previously reported FB factors^10^ that improve microvessel formation, TGFBI and HGF was significantly upregulated in FBs compared to PCs. Furthermore, when rHGF was added to the μVNs without FBs, there was an improvement in vessel anastomoses and a trend toward improved microvessel density. However, rHGF was not sufficient to fully reproduce the effect of FBs within the device and c-Met inhibition only partially reduced μVN formation. These data suggest that there are other factors important for μVNs formation. TGBI is induced by TGF-β1 and β2 and is a secreted extracellular matrix (ECM) protein involved in morphogenesis, adhesion/migration, tumorigenesis, wound healing, and inflammation. Clinically, mutations of this gene lead to corneal dystrophy. Interestingly, TGFBI has been described to inhibit cellular adhesion to ECM^25^ and may be important for EC tubulogenesis.^26^ However, addition of recombinant TGBI did not improve μVN formation (data not shown). Future studies will explore other differentially expressed factors of FBs and also combinations of these factors to more completely define the stimulatory effects of FBs.

An alternative explanation for the inability of PCs to support μVN formation in the absence of FBs is that the PC gene products may inhibit microvessel formation via cell–cell or cell–matrix interactions, yet still mediate modification of microvessel morphology. Several candidate proteins emerged from the RNA-seq data that could play a role in interactions between ECFCs and PCs. For instance, desmoplakin and several integrin subunits (*ITGA1*, *ITGA3*, *ITGA4*, *ITGA6*, *ITGA8*, *ITGA11*, *ITGAV*, and *ITGB5*) were significantly higher in PCs and could be involved in EC-PC cell junctions or PC-matrix interactions. More specifically, desmoplakin has been reported to interact with intermediate filaments and N-cadherin^27^ and is thought to be a key regulator of cell mechanics.^28^ Furthermore, N-cadherin is thought to be important for EC anchorage to PCs.^29,30^ In addition, integrins are heterodimeric proteins critical for cell–matrix interactions, mechanotransduction, and cell signaling.^31^ Consistent with our observation that ECs and PCs form a common basement membrane, several ECM proteins were upregulated in PCs that are known to be part of vascular basement membranes^32^ including collagen type IV isoforms, laminin chains, nidogen 1, SPARC, agrin, fibulin 2, and thrombospondin 1. In future studies, we will further investigate the critical factors that mediate the disparate interactions between ECs, FBs, and PCs.

The recent emergence of microfluidic technologies allows for improved control of complex cellular interactions and fluid flows at the micro-scale. In addition, microfluidic devices have low costs, are readily visualized in real-time, and the matrix and cellular components within them can be readily modified. The 3D orientation and perfusability of the microvessels formed within microfluidic devices allow investigators to observe cell–cell interactions and to test microvessel characteristics in ways that cannot be accomplished in traditional 2D monolayer or co-culture systems. Of note, microfluidic devices like the ones used here have previously been useful for investigation of both sprouting angiogenesis and μVN self-assembly as examined here.^33^ Sprouting angiogenesis can be accomplished by seeding ECs into the media channels of the device and allowing sprouting from the pores. The ECs sprout from outside and then into the hydrogel. We have observed that seeding ECs within the hydrogel to facilitate self-assembly generates perfusable μVNs more rapidly, and the microvessels traverse the entirety of the hydrogel. The self-assembled μVNs anastomose with the upper and lower pores to allow effective inflow and outflow of the media.

Interactions between ECs and PCs are clearly distinct from ECs and FBs. Furthermore, our electron microscopy studies appear to show the dynamic interaction and the formation of a common basement membrane between PCs and ECFCs. These images are reminiscent of reported TEM images of ECFCs and PCs in vivo.^34^ To our knowledge this is the first report demonstrating this process at the ultrastructural level within microfluidic devices. The observation of microfilaments and protrusions from these cells towards each other suggests a dynamic interaction that will need to be investigated further. The techniques used offer important experimental systems and tools for a deeper understanding of the interactions between the different cell types required for assembly of μVNs.

There is significant interest in the roles that FBs, PCs, and ECs play in tissue engineering approaches to improve microvessel formation and to model the microvasculature in pathology. A few examples of how our research findings fit into the broader field of tissue engineering and microvascular biology are worth briefly discussing. Investigators have demonstrated that human placental PCs alter the composition of extracellular matrix deposition with models of inflammation in vitro and suggest that these changes can alter EC adhesion molecule expression and neutrophil recruitment.^35^ The system that we have described here models in vivo microvasculature better than static culture systems because it incorporates both PC investment and perfusion. It could be used to further examine the effects of inflammation on immunological responses, cell adhesion, and extravasation under conditions of flow and microvessel mural cell coverage. Other investigators have proposed that co-cultures of ECs, PCs, and FBs on nanografted substrata can be used to understand how cells interact with the microenvironment to promote angiogenesis.^36^ Our system allows for both cellular self-assembly and matrix deposition, but simple modifications to the hydrogel composition could be added to further investigate how different microenvironments influence microvessel assembly and behavior. Another group has recently described how NG2^+^-PCs could be isolated from mice genetically modified to mark PC populations with a fluorescent protein (DsRed) used to sort cells from dissociated embryonic tissue.^37^ Part of the validation of these cells as PCs was the ex vivo incorporation into the blood vessels of explanted embryonic mouse skin. While this approach is quite convincing, we would argue that a simpler test of PC identity in vitro is to incorporate the cells into the microfluidic model system that we describe here. Alignment of the cells with EC-lined microvessels strongly argues for a PC identity given that tight association with ECs is a defining characteristic of PCs. This localization is likely a better indicator of identity than commonly used surface markers that can be variable and potentially overlap with other cell types.

While several new insights and potential future investigations have been revealed by the studies described here, there are some limitations that should be considered. Because these studies were carried out using primary cells derived from several different donors for ECFCs, PCs, and FBs, we have noted some variation in the morphology of microvessels within the microfluidic devices. For instance, EC vessel densities, diameters, and anastomoses can vary somewhat from experiment to experiment. Therefore, we have been careful to control the experiments and to include our positive control (co-culture ECs with high concentrations of FBs) with each study. Vessel anastomoses are based upon visual inspection of the tiled devices, however, a more accurate (and time-consuming) assessment of perfusability would be to flow fluorescent beads through each device and verify flows through each inlet and outlet pore. In addition, while our TEM studies have shown highly detailed images of the ultrastructural aspects of EC-PC interactions within the devices, it has been difficult to generate sample sizes needed for statistical analyses because of the complexity of the TEM processing and scarcity of definitively iron-labeled cells (FBs and PCs). An additional limitation is that iron labeling appears to be finite temporally, so we have thus far been unable to extend our time-course to observe how the EC-PC interactions appear at culture times beyond 7 days. In future studies, FBs and PCs could be labeled with different size iron particles to distinguish them so that the interactions between all three cell types could be examined at the ultrastructural level within the microfluidic devices.

In conclusion, we provide new tools and approaches for examining interactions of multiple cell types in a perfusable system that can be used to better inform the design and implementation of tissue-engineered μVNs in the future. Specifically, both FBs and PCs are likely necessary for tissue engineering of physiological human μVNs because these stromal populations provide different functions. Each cell type has a distinct phenotypic and transcriptional profile, which argues that they are not interchangeable in microvascular tissue engineering. FBs support formation and survival of μVNs whereas PCs have an important structural role.

## Methods

### Primary cells, fluorescent labeling, and protein quantification

ECFCs were cultured in EGM-2MV (Lonza, Walkersville, MD, USA) on gelatin (Sigma-Aldrich, St. Louis, MO, USA)-coated plates and isolated from discarded and de-identified human umbilical vein cord blood as “late outgrowth” cells, as previously described.^4,38^ Human microvascular placental PCs were isolated from discarded and de-identified placentas as explant-outgrowth cells, also as previously described.^12^ Human lung FBs were purchased from Lonza. Both FBs and PCs were serially cultured in Medium 199 (Gibco, Grand Island, NY, USA) plus 20% FBS, 2 mM L-glutamine, 100 U/ml penicillin, and 100 μg/ml streptomycin (all from Invitrogen, Carlsbad, CA, USA). All cells were used between subculture 2–15.

Where indicated, cells were transduced with lentivirus (rLV.EF1.mCherry-9 or rLV.EF1.AmCyan1-9) to induce expression of mCherry or AmCyan per the vendor’s recommended protocol (Vectalys, Toulouse, France). Multiplicity of Infection (MOI) for mCherry ECFC and AmCyan PCs and FBs were 7 and 50.

For quantification of HGF secretion, 5.0 × 10^5^ PCs and FBs were grown in a single well of a 24-well dish for 24 h and enzyme-linked immunosorbent assay (ELISA) was performed per manufacture’s protocol (R&D Systems, Minneapolis, MN, USA). To test whether co-culture of ECFCs could affect HGF production in PCs and FBs, 5.0 × 10^5^ ECFCs were grown in a cell culture transwell insert (Fisher Scientific, Pittsburgh, PA, USA) with 0.4 μm pore sizes. Inserts were positioned above 5.0 × 10^5^ PCs or FBs plated onto the bottom of the cell culture well. Media was collected after 24 h for HGF ELISA.

### Microfluidic device set up

The microfluidic devices contain six ports for loading cells, matrix, and media (AIM Biotech, Singapore). The undersides of the devices have permeable laminates that facilitate gas exchange while μVNs are being cultured. Each device contains a central channel (10.5 mm long and 1.3 mm wide) and two flanking media channels that are 0.5 mm wide. For more detailed information about microfluidic devices, see Supplementary Figure 1. Central channels were loaded with cells and 2 mg/mL fibrinogen after addition of bovine thrombin (2 U/ml) (Sigma). After polymerization, EGM-2MV media was loaded into top channels. To change the media, the top two media wells were filled with 70 and 50 μL of media, left to right, respectively, and the bottom two media wells were filled with 30 μL of EGM-2MV. Media in the wells were changed twice a day for the first 3 days, and then once a day for the rest of the duration of the experiment. To test effects of HGF on μVN formation, recombinant HGF (rHGF) (R&D Systems) or the HGF receptor (c-Met) inhibitor INCB28060 (Cayman Chemicals, Ann Arbor, MI, USA) at indicated concentrations was added to the media loaded into the microfluidic devices.

### Microscopy

For quantification of vessel densities, an epifluorescence microscope (Leica DMI6000, Wetzlar, Germany) was used to image the devices. Multiple individual images were tiled together to form composite images of the entire gel channel. For confocal imaging, a Leica TCS SP-5 Confocal Microscope was used. For time-lapse video images, a Zeiss Axiovert 200 M inverted fluorescence microscope with a Hamamatsu ORC-AG high-resolution camera and Volocity imaging software (PerkinElmer, Waltham, MA, USA) was used to capture flow of Sphero^TM^ 2 μm high intensity fluorescent beads (Spherotech, Lake Forest, IL, USA). For association studies, association was defined as direct contact between an AmCyan FB or AmCyan PC and an mCherry labeled microvessel. Fields of interest were visualized with confocal microscopy. Z-stacks of serial sections were generated to capture the entire interaction and depth of the vessel. The 3D reconstruction of the vessel was analyzed for the presence or absence of cell–cell association or PC alignment along the microvessel.

For immunofluorescence (IF) studies, microfluidic devices were fixed and permeabilized as previously described^13^ with extension of incubation times to allow for perfusion of the microfluidic devices. Prior to visualization, protocol steps required perfusion of devices via inlet and outlet pores as described for media loading. Fixation and permeabilization steps were performed for 30 min, primary antibody incubations were done at 4 °C overnight, and secondary incubations were done for 2 h. To identify human ECs, Ulex Europaeus Agglutinin I DyLight 649 (Vector Laboratories, Burlingame, CA, USA) (1:100) was used. For collagen IV staining, rabbit polyclonal antibody to collagen type IV antibody (1:100) (Sigma) was used. As the secondary antibody for collagen IV, Alexa Fluor 594 Goat Anti-Rabbit IgG (1:100) (Invitrogen) was used.

For transmission electron microscopy, PCs and FBs were pre-labeled by incubating cells with 50 μg/ml of Molday ION Rhodamine B (BioPal, Worcester, MA, USA) for 18 h in a single well of a 6-well plate. After the incubation period and three washes, cells were loaded into devices and μVNs were established and perfused for 7 days before being fixed in 2.5% glutaraldehyde in 0.1 M sodium cacodylate buffer (pH 7.4) for 1 h at room temperature. The devices were then rinsed in cacodylate buffer through the microfluidic device perfusion ports. At 1 h post fixation, 1% osmium tetroxide was added, followed by rinsing and en-bloc staining in 2% aqueous uranyl acetate for an additional hour. Samples were then rinsed and dehydrated in an ethanol series. LRWhite (Electron Microscopy Sciences, Hatfield, PA, USA) acrylic resin was used to infiltrate the sample overnight followed by 48-h incubation at 60 °C. LRWhite was chosen over conventional epon resins because of its low viscosity that allowed for better flow into the thin microfluidic channels. Hardened blocks were cut using a Leica UltraCut UC7. Next, 60 nm sections were collected on formvar/carbon coated copper slot grids and contrast stained using 2% uranyl acetate and lead citrate. Samples were viewed on FEI Biotwin TEM at 80 Kv. Images were captured on Morada CCD and iTEM (Olympus) software. For EM tomography, 250 nm thick sections were collected using FEI Tecnai TF20 at 200 Kv with 15 nm fiducial gold to aid in alignment. Data were collected with SerialEM on a FEI Eagle 4 × 4 CCD camera using tilt angles of −60 to 60° and reconstructed using IMOD. The 3D model was constructed using 3dmod software package in IMOD, following general modeling protocol. The slices within tomogram were manually drawn and contoured to generate a precise 3D reconstruction of the imaging sections.^39,40^

### Quantification of microvessel characteristics

To calculate the microvessel density, we developed a MATLAB code with a graphical user interface (GUI). The GUI was used to set parameters to filter out objects that were not microvessels (such as single cells). A gray threshold was used to create binary images. Single cells (which are smaller and rounder) were filtered out by setting a threshold on the minimum number of pixels as well as eccentricity of connected regions. Other regions (such as debris) were removed by manually drawing a region or targeting small areas for deletion. The vessel density was calculated as the number of pixels above threshold divided by the image area selected. This value is expressed as a decimal fraction. The MATLAB code, files, and sample analysis (Supplementary Figure 2) are included in the Supplementary Materials. For analysis of anastomoses, tiled images were visually inspected for mCherry labeled-EC vessel formation at the pore of the microfluidic devices. Values are expressed as a decimal fraction of the total number of pores within the devices (total pore number is 54). For analysis of vessel diameters, FIJI software^41^ with the Vessel Analysis plugin was used per detailed instructions for the Diameter Measurement function. Values derived are the mean diameters of the μVNs examined.

### Statistical analysis

Statistical analysis was performed with Prism 7.04 (GraphPad) using two-way ANOVA with post hoc Bonferroni corrections when measuring network densities across multiple days. When multiple groups were compared at single time points, one-way ANOVA was carried out, but when only two groups were compared, unpaired two-tailed student’s *t*-tests were performed. Three devices or samples were analyzed per condition. Graphs are presented as means with standard deviations.

### RNA-seq analysis

Confluent FBs from three different donors for each cell type were grown in one well of a 12-well plate and total RNA was purified using the RNeasy Mini Kit (Qiagen) with an on-column DNase treatment. Preparation and sequencing were performed as described previously for PC RNA-seq analysis.^19^ For purified total RNA collected from FB samples, the three strand-specific sequencing libraries were produced following the Illumina TruSeq stranded protocol. According to Illumina protocol, the libraries underwent 76-bp paired-end sequencing using an Illumina HiSeq 2500, generating an average of 32 million paired-end reads per library. Both the original PC^39^ and the new FB sequences were processed together through the same analysis pipeline. For each read, the first 6 and the last nucleotides were trimmed to the point where the Phred score of an examined base fell below 20 using in-house scripts. If, after trimming, the read was shorter than 45 bp, the whole read was discarded. Trimmed reads were mapped to the human reference genome (hg38) with HISAT2 v2.1.0^42^ indicating that reads correspond to the reverse complement of the transcripts and reporting alignments tailored for transcript assemblers. Alignments with quality score below 20 were excluded from further analysis. Gene counts were produced with StringTie v1.3.3b^43^ and the Python script “prepDE.py” provided in the package. StringTie was limited to assemble reads matching the reference annotation GENCODE v27.^44^ After obtaining the matrix of read counts, differential expression analysis was conducted and normalized counts were produced using DESeq2.^45^ *P*-values were adjusted for multiple testing using the Benjamini–Hochberg procedure.^46^

### Ethics

Protocols were reviewed by the Yale Institutional Review Board (IRB) (ID 2000025795) and it was determined that experiments described are not consider human subject research and thus do not require IRB approval.

### Preprint

A significant portion of this work has been previously deposited as a preprint as, Kosyakova et al. Differential functional roles of fibroblasts and pericytes in the formation of tissue-engineered microvascular networks in vitro, at 10.1101/558841.

### Reporting summary

Further information on research design is available in the Nature Research Reporting Summary linked to this article.

## Supplementary information

Supplementary Figures and Table

Supplementary Data 1

Supplementary Movie 1

Supplementary Movie 2

Supplementary Data 2

Supplementary Data 3

Supplementary Data 4

Reporting Summary Checklist

## Data Availability

Representative images, analyses, code, and expression data are included in this published article (and its Supplementary information files). Sequencing data for the FB samples were deposited in NCBI’s Gene Expression Omnibus (GEO) under accession number GSE122389. Raw image files are available from the corresponding author upon reasonable request.
