# Supplementary material for: Differential functional roles of fibroblasts and pericytes in the formation of tissue-engineered microvascular networks in vitro
Source: NPJ Regen Med. 2020 Jan 6;5:1. doi: 10.1038/s41536-019-0086-3 (PMC6944695; doi:10.1038/s41536-019-0086-3)
Supplement: Supplementary file 1 — Supplementary Figures and Table [file 41536_2019_86_MOESM1_ESM.pdf]

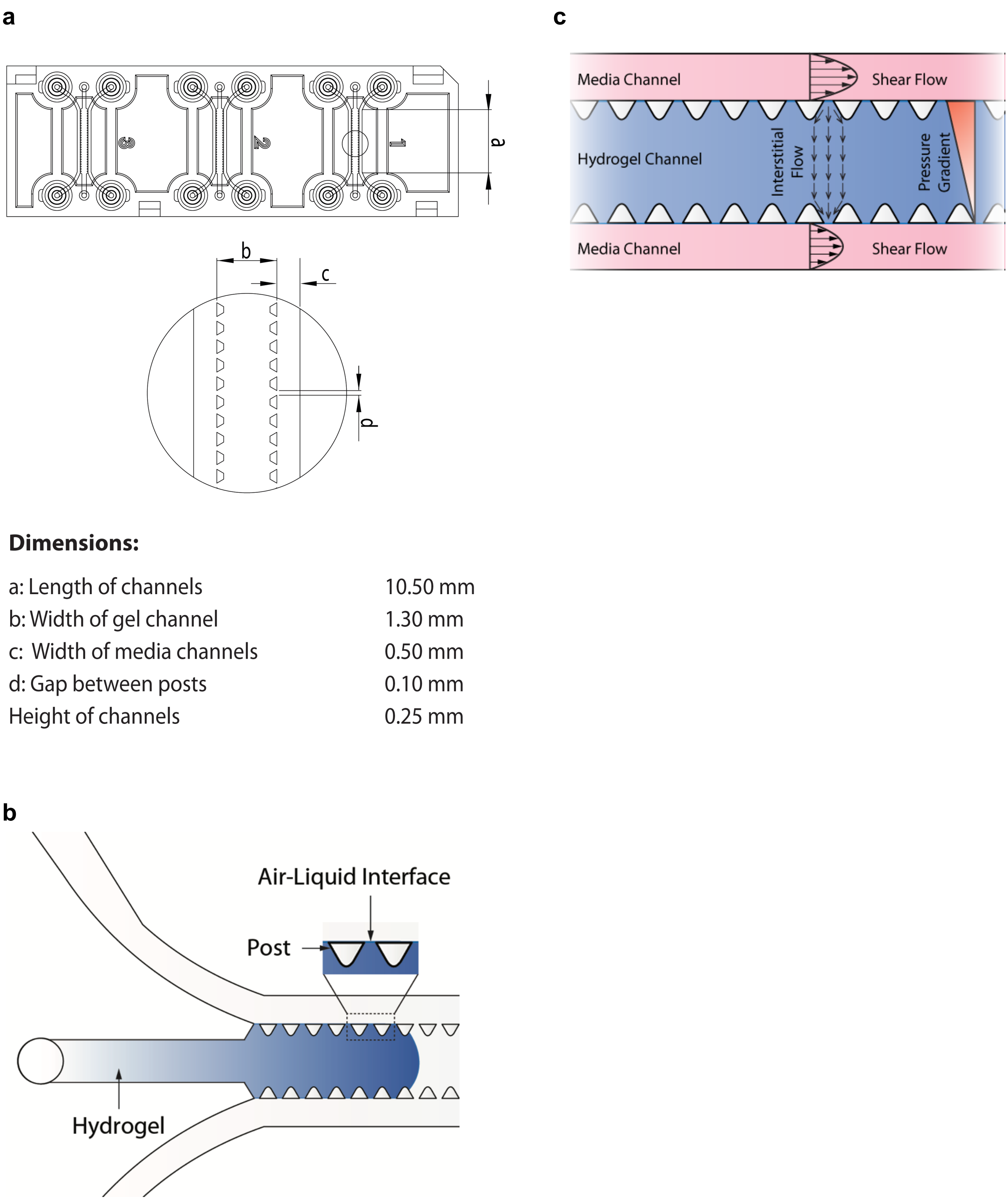

Supplementary Figure 1. Microfluidic device design. (a) Schematic diagram of AIM Biotech microfluidic devices. (b) Illustration of how microfluidic devices are loaded with hydrogels in the central channel. (c) Illustration of how microfluidic flows move from inlet pores, through the interstitium of the hydrogel, and through the outlets. Permission granted by AIM Biotech to reproduce these images.

```
clear all
close all

images = dir('*cut.tif');

% parameters found in GUI:
thresh       = 0.1; % for binarizing image
pixel_thresh = 500; % minimum number of pixels for counting a region as vessel
ecc_thresh   = 0.4; % minimum threshold to exclude round structures

mark_objects                = 1; % possibility to mark and remove objects manually
draw_boarder_around_objects = 1; % possibility to draw boarder around regions to exclude

for i = 1:length(images)

    img_name   = images(i).name;
    img        = imread(img_name);     % load in image (4 channels)
    img        = img(:,:,1);           % only first channel is important
    img_height = size(img,1);
    img_width  = size(img,2);

    figure(1)
    imshow(img)

    img_bw       = imbinarize(img,thresh); % binarize image
    img_cr       = bwconncomp(img_bw); % find connected regions
    numPixels    = cellfun(@numel,img_cr.PixelIdxList); % number of pixels for each connected region
    eccentricity = regionprops(img_cr,'Eccentricity'); % eccentricity for each connected region
    ecc          = struct2cell(eccentricity);

    % filter out objects which are not vessels based on their size and
    % eccentricity
    img_detected_vessels = zeros(img_height, img_width);
    for j = 1:img_cr.NumObjects
        if numPixels(j) > pixel_thresh && ecc{1,j} >= ecc_thresh
            img_detected_vessels(img_cr.PixelIdxList{j}) = 1;
        end
    end

    figure(2)
    imshow(img_detected_vessels)

    %remove isolated objects manually:
    if mark_objects == 1
       index = 1;
       h = msgbox('Mark region')
       [x,y] = ginput(1);
       m = round(y);
       n = round(x);
       linindex = sub2ind([img_height, img_width],m, n);
       positions_list(index) = linindex;

       choice = questdlg('Mark another region?','Message box', 'Yes', 'No','Yes')
       while strcmpi(choice,'Yes');
             index = index + 1;
             [x,y] = ginput(1);
             m = round(y);
             n = round(x);
             linindex = sub2ind([img_height, img_width],m, n);  % conversion of x,y coordinates into linear indices
             positions_list(index) = linindex;
             choice = questdlg('Mark another region?','Message box', 'Yes', 'No', 'Yes')
       end

       for k = 1:img_cr.NumObjects   % remove all marked regions
           exist = [];
           compare = ismember(linindex,img_cr.PixelIdxList{k});
           exist = find(compare);
           if length(exist)~= 0
               img_detected_vessels(img_cr.PixelIdxList{k}) = 0;
           end
       end
    end

    % remove objects connected to vessels by drawing a boarder:
    if draw_boarder_around_objects == 1
        index = 1;
        h = msgbox('Select region')
        selected_region{index} = roipoly();
        choice = questdlg('Select another region?','Message box', 'Yes', 'No','Yes')
        while strcmpi(choice,'Yes');
              index = index + 1;
              selected_region{index} = roipoly();
              choice = questdlg('Select another region?','Message box', 'Yes', 'No', 'Yes')
        end
        selected_regions = selected_region{1};
        for l = 2:length(selected_region)
            selected_regions = selected_regions + selected_region{l};
        end

        img_detected_vessels = img_detected_vessels-selected_regions;
    end

    figure(2)
    imshow(img_detected_vessels)

    vessel_density(i) = nnz(img_detected_vessels)/(img_height*img_width);
end
```

Supplementary MATLAB Code. Microvessel analysis.

a

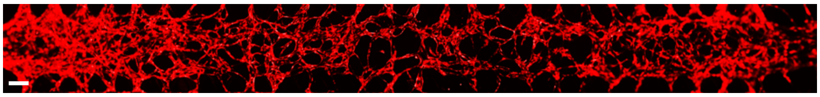

b

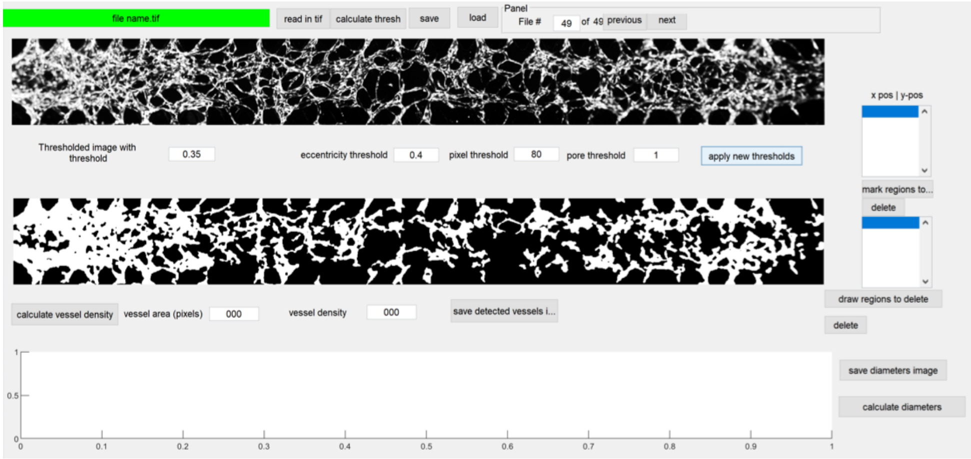

Supplementary Figure 2. Microvascular density quantification. Example of tiled image of μVNs uploaded into the MATLAB program. Graphical user interface (GUI) was used to set thresholds for vessel density calculations.

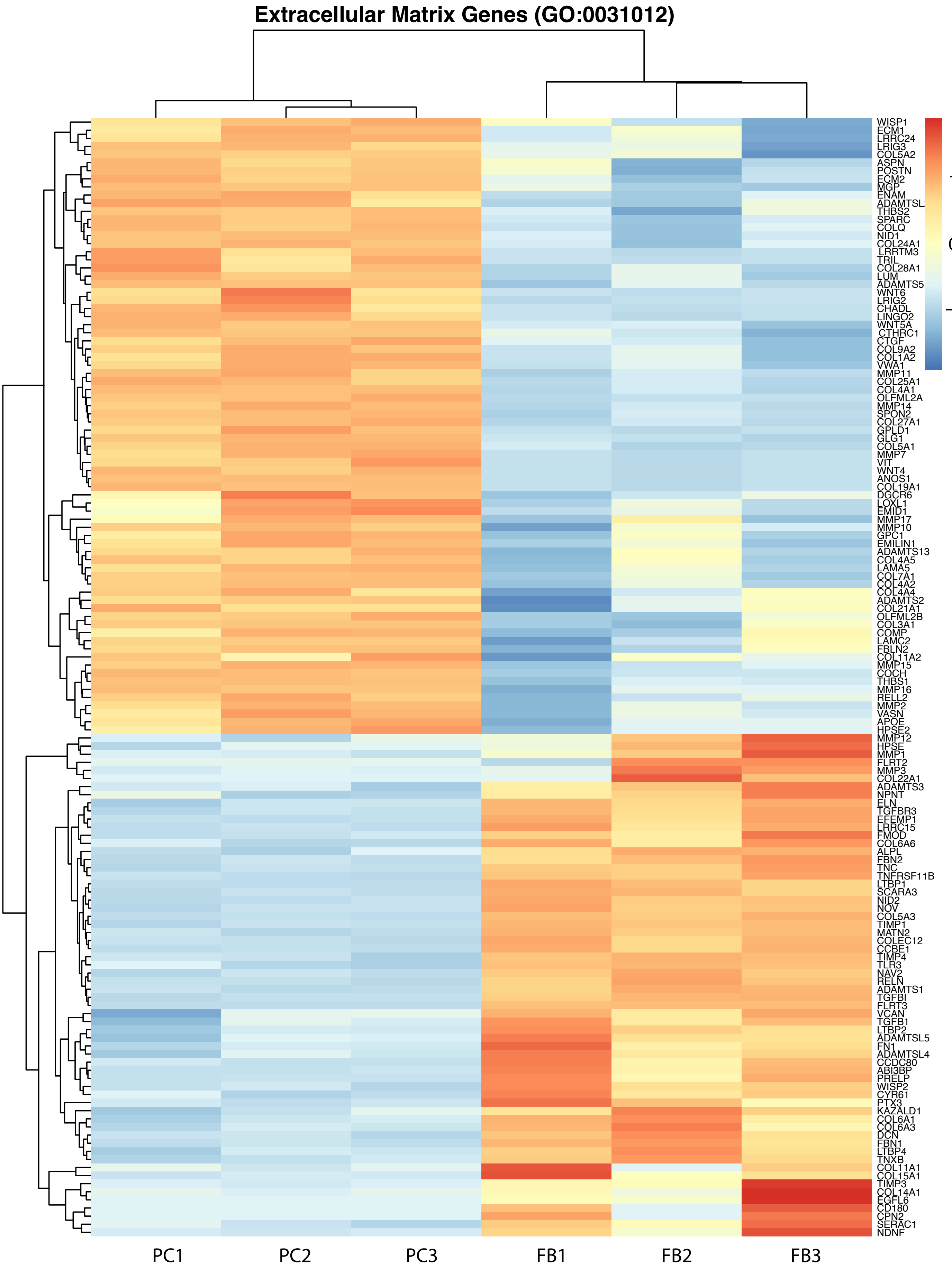

Supplementary Figure 3. Heatmap of PC and FB expression of extracellular matrix genes

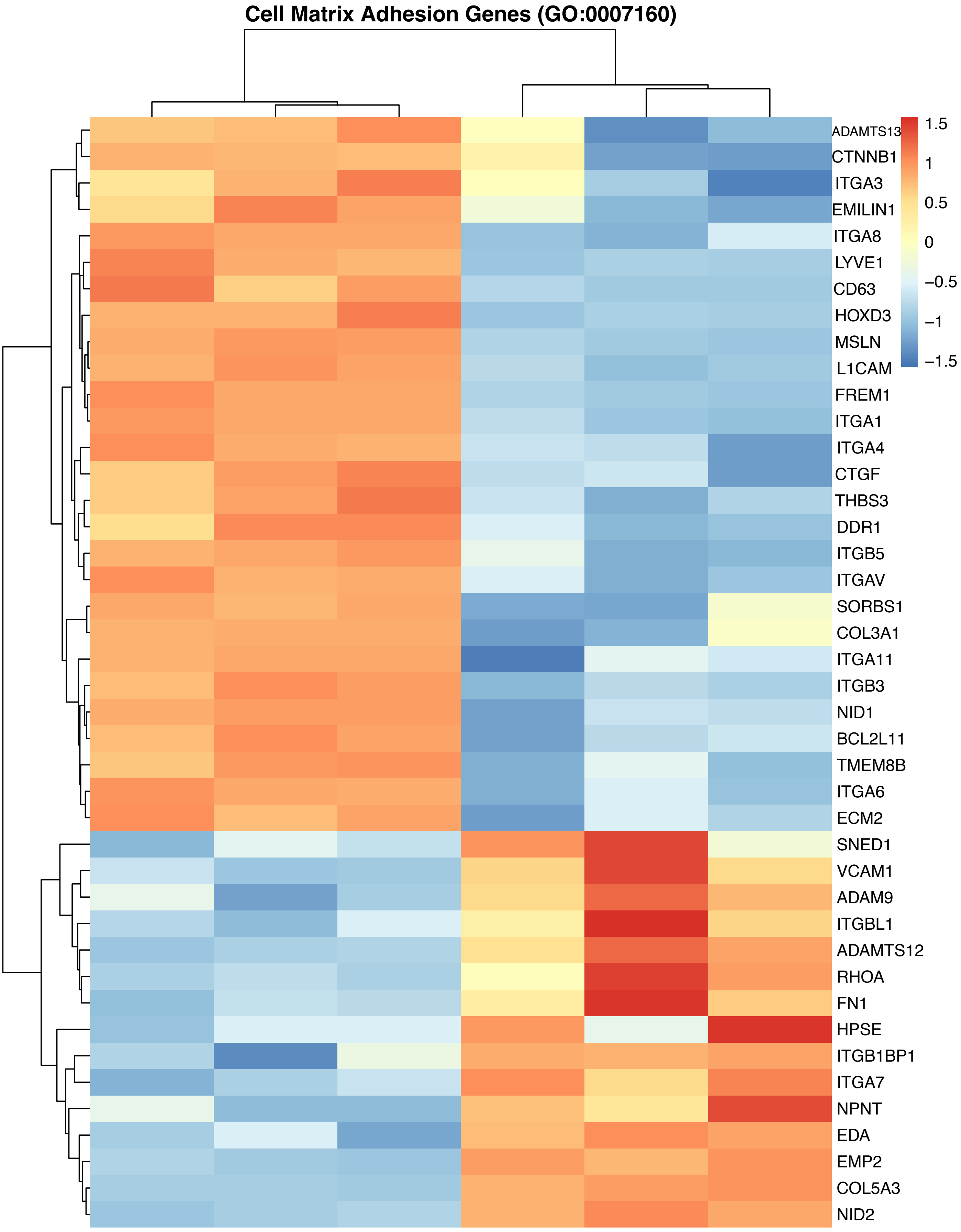

Supplementary Figure 4. Heatmap of PC and FB expression of cell matrix adhesion genes.

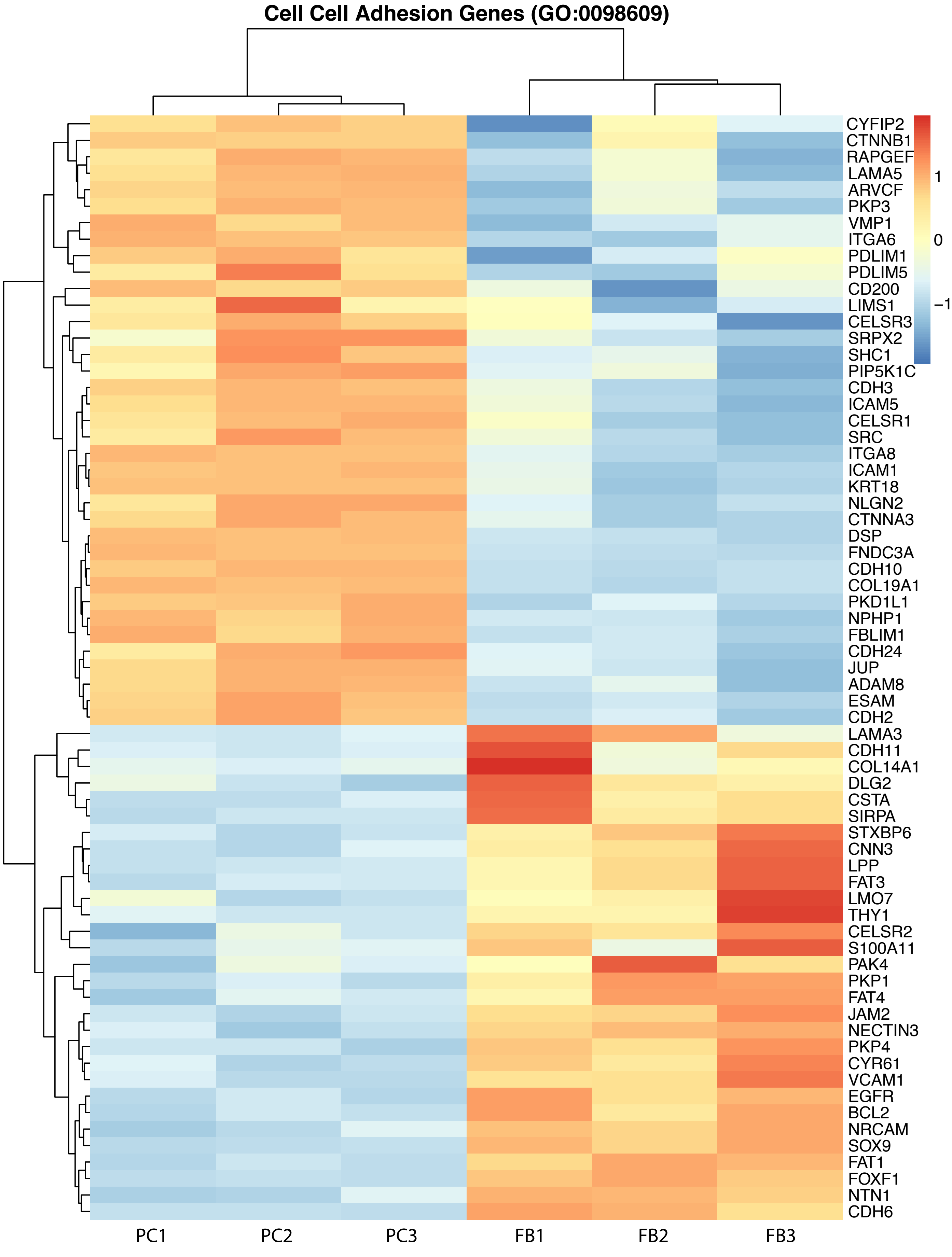

Supplemental Figure 5. Heatmap of PC and FB expression of cell cell adhesion genes

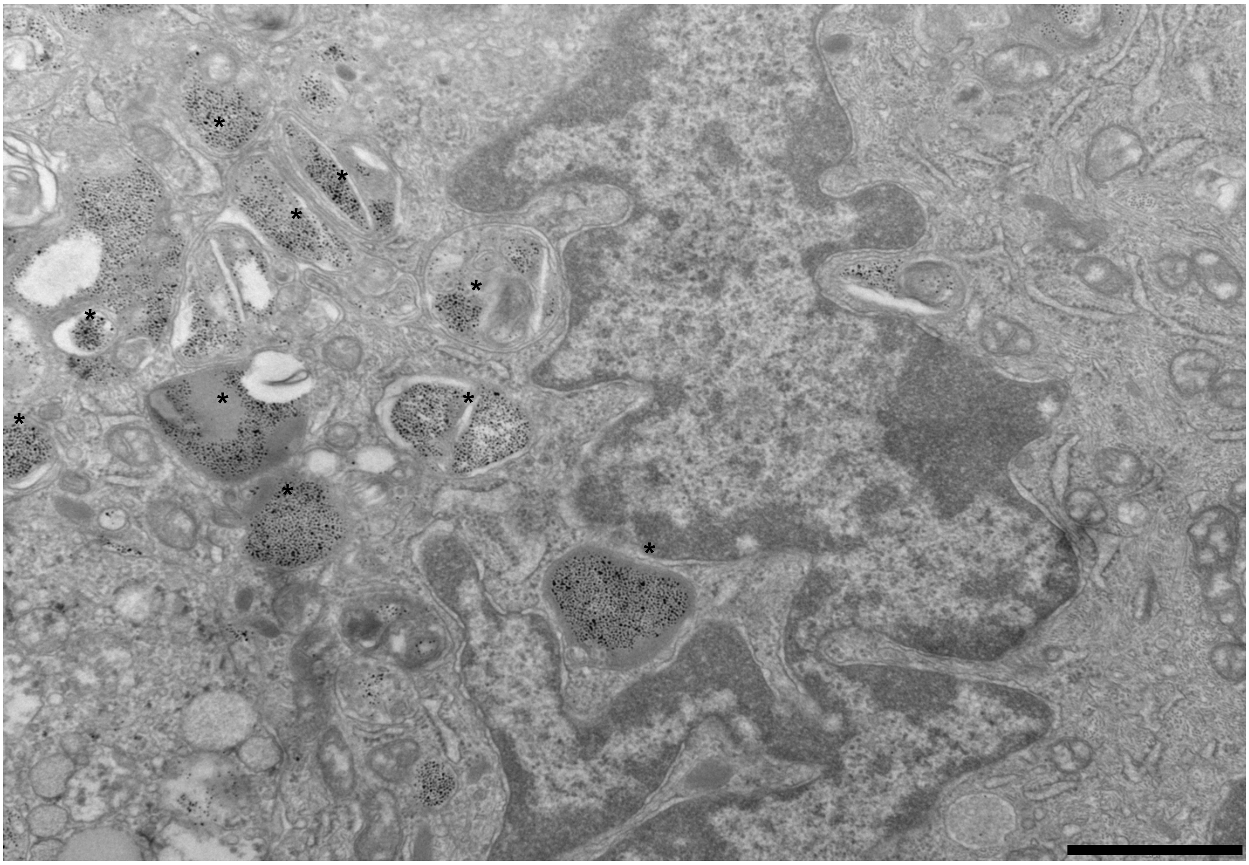

Supplementary Figure 6. Iron-labeled PC. PC incubated with iron MION particles and processed for TEM after 5 days. Asterisks (*) indicated iron-labeled endosomes.

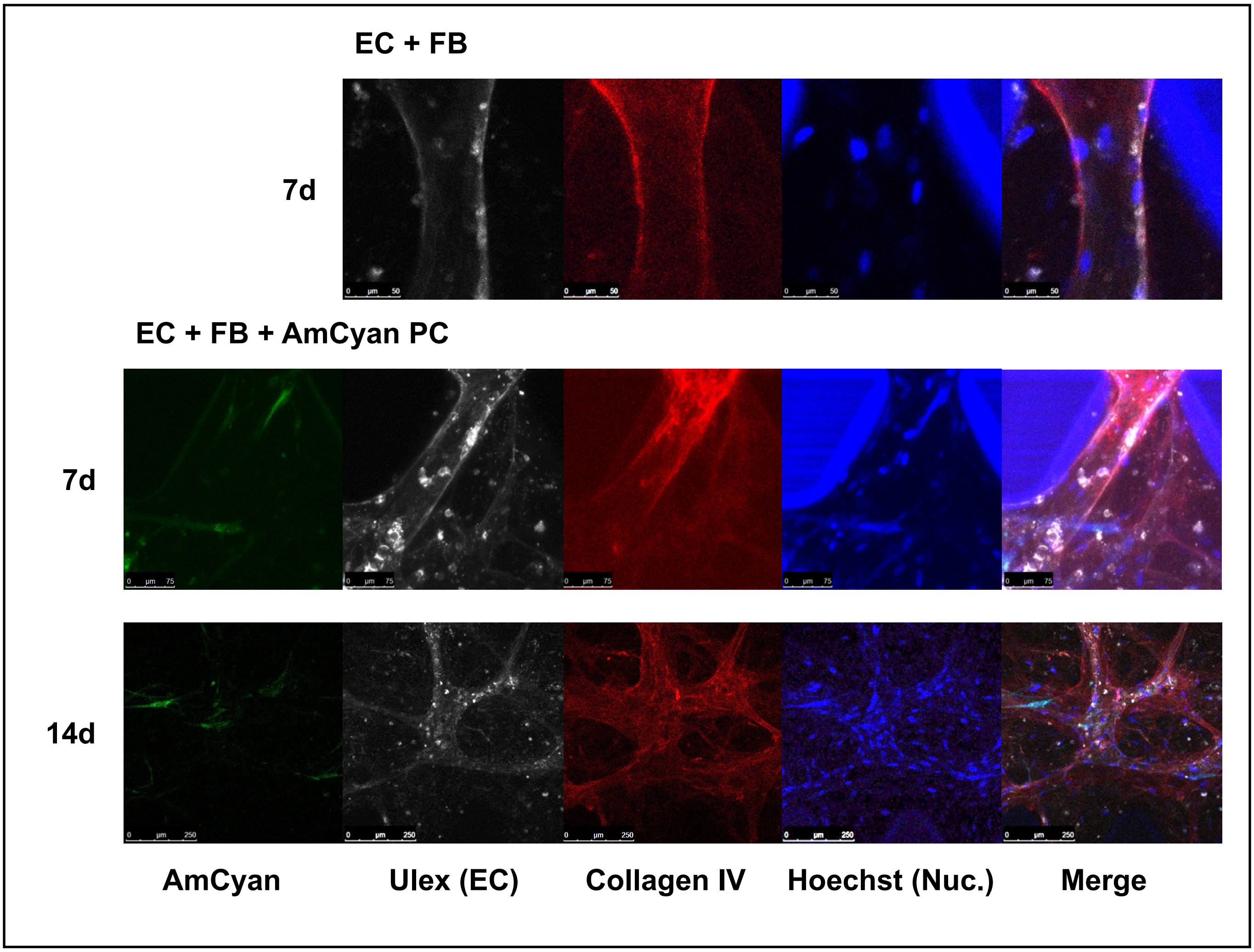

Supplementary Figure 7. Collagen IV staining of μVNs. (Top) Confocal images of EC and FB co-cultured within microfluidic device for 7 days. (Middle) EC, FB, and AmCyan expressing PC co-cultured for 7 and 14 days. ECs, collagen IV, and nuclei were stained with Ulex, anti-collagen IV, and Hoechst. Specific scale bars are labeled within each panel.

Supplementary Data 1. Transcriptional profiling of PCs and FBs. Gene expression data from RNA-seq comparison of human placental PCs and human lung FBs.

Supplementary Data Files 2-4. **MATLAB microvessel analysis code.**

Supplementary Movie 1. Perfusion through µVNs. High intensity fluorescent beads (2 µm) were perfused through µVNs to confirm perfusability.

Supplementary Movie 2. iMOD 3D Modeling of EC and PC interaction. EC border (in green), PC border (in blue), and microfilaments between the cells were manually contoured and meshed to construct a 3D model. The model was constructed in 3dmod, an image processing dialog found under iMOD programs. The tomography slices were opened in 3dmod and the outline of the cells was modeled in the main 3dmod window as a closed object. The microfilaments were modeled using the slicer tool which allowed us to follow and adjust for the x, y, and z orientation of the filaments. The contours were then meshed together and viewed in the model window. To better distinguish the origin of the microfilaments located between cells, microfilaments were outlined in three colors: dark blue, yellow, and magenta. Dark blue and yellow filaments originate in PC and EC, respectively. Magenta filaments connect EC and PC. Scale bar is 200 nm.

Cell-cell Genes_PC	Cell-cell Genes_FB	Cell-matrix Adhesion Genes_PC	Cell-matrix Adhesion Genes_FB	Extracellular Matrix Proteins_PC	Extracellular Matrix Proteins_FB
NLGN2	CDH6	ITGA11	ADAMTS12	ANOS1	LTBP1
CD200	JAM2	ECM2	EDA	COL11A2	CPN2
CDH10	SIRPA	SORBS1	ITGB1BP1	ASPN	LRRC15
ADAM8	FOXF1	ITGA3	NPNT	ECM2	TIMP4
CTNNA3	LMO7	ITGA1	RHOA	WNT5A	COLEC12
PIP5K1C	CDH11	MSLN	HPSE	COL4A1	TNFRSF11B
CXADR	NRCAM	HOXD3	COL5A3	COL4A2	NOV
PKP3	BCL2	ITGB5	ADAM9	LAMB1	KAZALD1
NPHP1	PKP4	BCL2L11	ITGBL1	LRRTM3	CCDC80
SRPX2	SOX9	L1CAM	EMP2	MMP14	EFEMP1
PDLIM5	COL14A1	ITGAV	ITGA7	COL4A5	ALPL
VMP1	PKP1	ADAMTS13	SNED1	VASN	COL15A1
SRC	CSTA	ITGA4	NID2	DGCR6	CCBE1
RAPGEF1	NTN1	CTNNB1	VCAM1	TRIL	TNXB
CDH24	CYR61	EMILIN1	FN1	VIT	NPNT
KRT18	STXBP6	COL3A1		LRRC24	COL14A1
DSP	THY1	ITGA6		LAMA5	FBN2
JUP	EGFR	TMEM8B		COL28A1	ABI3BP
CTNNB1	CNN3	ITGA8		COL27A1	ADAMTS1
ITGA6	NECTIN3	ITGB3		ENAM	FGF10
EPCAM	PAK4	LYVE1		EMID1	HPSE
ITGA8	S100A11	CTGF		ADAMTS5	MATN2
ARVCF	FAT1	CD63		POSTN	VCAN
ICAM1	VCAM1	FREM1		MMP15	HSPG2
CDH2	DLG2	NID1		VWA1	WISP2
SYNJ2BP-COX16		THBS3		COL1A2	NAV2
ICAM5				MMP16	RELN
COL19A1				THBS1	TGFBI
FNDC3A				ADAMTS10	COL5A3
CDH3				LOXL1	CD180
PKD1L1				WNT4	COL6A1
ESAM				THBS2	COL6A3
CYFIP2				MMP2	TIMP3
PDLIM1				SPON2	ADAMTS3
LRRC7				ADAMTS13	TGFBR3
FBLIM1				GLG1	DCN
SHC1				MGP	EGFL6
				COMP	FLRT2
				LTBP3	FLRT3
				MMP11	FMOD
				EMILIN1	PRELP
				GPC1	LTBP2
				ADAMTSL2	FGFR2
				APOE	HNRNPM
				COL7A1	CYR61
				COL3A1	TNC

				**LRIG2**	**LTBP4**
				**LUM**	**TLR3**
				**COL5A2**	**NID2**
				**COLQ**	**COL22A1**
				**CTHRC1**	**MMP1**
				**COCH**	**MMP3**
				**PLSCR1**	**FBN1**
				**COL5A1**	**MMP12**
				**GPLD1**	**ELN**
				**FBLN2**	**TIMP1**
				**WISP1**	**ADAMTSL5**
				**LINGO2**	**ADAMTSL4**
				**HPSE2**	**COL6A6**
				**SPARC**	**SERAC1**
				**MFAP4**	**CLU**
				**COL19A1**	**MYOC**
				**COL24A1**	**TGFB1**
				**LRIG3**	**NDNF**
				**COL25A1**	**COL11A1**
				**CTGF**	**SCARA3**
				**RELL2**	**PTX3**
				**COL21A1**	**FN1**
				**AGRN**	
				**COL9A2**	
				**ADAMTS2**	
				**COL4A4**	
				**ADAMTS4**	
				**MMP7**	
				**OLFML2A**	
				**RARRES2**	
				**WNT2**	
				**OLFML2B**	
				**MMP10**	
				**CHADL**	
				**ECM1**	
				**EGFL7**	
				**NID1**	
				**WNT6**	
				**MMP17**	
				**LAMC2**	

Supplementary Table. Genes included in GO analyses. List of extracellular matrix, cell-matrix adhesion, and cell-cell adhesion gene subsets examined by GO analyses.
